# The occurrence of coronary artery lesions in Kawasaki disease based on C-reactive protein levels: a retrospective cohort study

**DOI:** 10.1186/s12969-021-00566-6

**Published:** 2021-06-02

**Authors:** Hyo Soon An, Gi Beom Kim, Mi Kyoung Song, Sang Yun Lee, Hye Won Kwon, Joo Won Lee, Eun Jung Bae

**Affiliations:** Department of Pediatrics, Seoul National University Children’s Hospital, Seoul National University College of Medicine, 101 Daehak-ro, Jongno-gu, Seoul, 03080 South Korea

**Keywords:** Acute-phase reactants, Coronary aneurysm, Intravenous immunoglobulins, Mucocutaneous lymph node syndrome, Risk factors

## Abstract

**Background:**

This study aimed to assess the occurrence of coronary artery lesions (CAL) in patients with Kawasaki disease (KD) according to serum C-reactive protein (CRP) levels.

**Methods:**

This retrospective analysis was based on the nationwide survey of KD conducted in the Republic of Korea between 2015 and 2017. We enrolled 9131 patients and defined low (< 3 mg/dL) and high (≥3 mg/dL) CRP groups. Demographic data, clinical characteristics, z-scores, and scores based on the Japanese criteria for CAL were compared between the two groups. Logistic regression analysis was used to identify CAL risk factors.

**Results:**

The low CRP group accounted for 23% of patients. The mean age at diagnosis was higher in high CRP group compared to the low CRP group (34.4 ± 24.9 vs 31.7 ± 24.8 months, *p* < 0.001). Fever duration before treatment was not significantly different between the two groups (5.1 ± 1.7 days vs. 5.2 ± 2.1 days; *p* = 0.206). A non-response to intravenous immunoglobulin treatment was found in 1377 patients (20.1%) and 225 patients (11.7%) in the high and low CRP groups, respectively (*p* < 0.001). CAL were found in 12.9 and 18.3% of the high and low CRP patients, respectively (*p* < 0.001), based on z-scores; and in 9.9 and 12.5%, respectively (*p* = 0.001), based on the Japanese criteria in the acute phase. The giant coronary artery aneurysm occurrence ratio was similar between groups (*p* = 1.0).

**Conclusions:**

CAL occurred in patients with both high and low CRP. Therefore, patients with KD should be carefully monitored regardless of their CRP levels.

**Supplementary Information:**

The online version contains supplementary material available at 10.1186/s12969-021-00566-6.

## Background

Kawasaki disease (KD) is a systemic inflammatory vascular disease that results in coronary artery complications in 25% of untreated patients [1]. This disease is reported in high frequency, particularly in Northeast Asia, including Korea, and occurs primarily in children under 5 years of age [2]. Systemic inflammation in KD is reflected by an elevation of serum C-reactive protein (CRP) levels, an acute phase reactant. In general, high serum CRP levels are expected in KD. Therefore, in patients presenting with incomplete KD that does not fulfill the diagnostic criteria, a CRP serum level > 3 mg/dL is used as a criterion to confirm KD [1].

At the same time, this rise in serum CRP levels is also used as an index to predict KD that is refractory to treatment [3–5]. Since the main purpose of KD treatment is to minimize the occurrence of coronary artery complications through appropriate treatment, patients with high CRP, which are expected to be less responsive to treatment, are treated with caution. However, in clinical practice, coronary artery complications are often seen even in KD patients with low CRP levels. In this study, we aimed to assess the clinical features and occurrence of coronary artery lesions (CAL) in patients with KD according to serum CRP levels. In addition, we tried to confirm whether CAL occurred in patients with low serum CRP level before IVIG treatment.

## Methods

This study was based on the 9th nationwide KD survey performed under the guidance of the Korean Society of KD in the Republic of Korea, which was conducted on patients with KD who visited a hospital between January 2015 and December 2017. Data were collected using a questionnaire regarding the clinical characteristics of patients, which were filled in by hospital medical staff. The survey was approved by the Institutional Review Board of our university hospital (no. H-1710-109-895, approved November 20, 2017).

The survey obtained data from 15,387 patients. Of these patients, 6256 were excluded due to missing data on serum CRP levels, coronary artery size, height, and weight. Eventually, 9131 patients were enrolled in the study and were divided into two groups according to their serum CRP levels: low (< 3 mg/dL) and high (≥ 3 mg/dL). We recorded serum CRP levels just before the start of acute treatment. Coronary artery evaluation was performed with echocardiography twice, during the acute phase (maximal dilatation of the coronary arteries after intravenous immunoglobulin [IVIG] treatment; *n* = 9014) and post-convalescent period (approximately 8 weeks after disease onset; *n* = 8206).

The diagnosis of complete KD was based on the presence of four or more of the principal clinical criteria (extremity changes, rash, conjunctivitis, oral changes, cervical lymphadenopathy), and patients with three or fewer criteria and compatible laboratory or echocardiographic findings were considered to have incomplete KD [1]. Non-responsiveness to initial IVIG treatment was defined as the requirement for the administration of second-line treatment, such as a second dose of IVIG or intravenous steroids.

The complications of KD were assessed based on the development of CAL on the left main coronary artery. The z-score was calculated using a previously reported formula [6], and a z-score ≥ 2.5 was considered to be CAL. We also assessed CAL development according to the criteria of the Japanese Ministry of Health and Welfare (Japanese criteria) [7, 8], which defined CAL as a coronary artery diameter ≥ 3 mm in children < 5 years old and ≥ 4 mm in children ≥5 years old. A z-score ≥ 10.0 and diameter > 8 mm were considered to indicate a giant coronary artery aneurysm (CAA).

### Statistical analysis

Data were expressed as the mean ± standard deviation, median (range), or percentage (%), as appropriate. We compared the characteristics of patients, treatment, and coronary artery complications between the low and high CRP groups. An independent t-test was performed to compare continuous variables, and a chi-squared test was used to analyze categorical variables. We performed logistic regression analysis to identify potential risk factors for the development of CAL. A *p*-value < 0.05 was considered statistically significant. All statistical analyses were conducted using IBM SPSS Statistics for Windows, version 20 (IBM Corp., Armonk, NY, USA).

## Results

The mean age at diagnosis was 33.7 ± 24.9 months, and the proportion of male children was 58.1% (male:female ratio, 1.41:1). The average duration of fever before treatment was 5.1 ± 1.8 days, and the total fever duration was 6.5 ± 2.3 days. The mean serum CRP level in all children was 7.4 ± 5.8 mg/dL. The low CRP group accounted for 23% of all KD patients.

### Comparison of clinical characteristics by CRP level

The mean age at diagnosis was higher in the high CRP group compared to the low CRP group (34.4 ± 24.9 vs 31.7 ± 24.8 months, *p* < 0.001) and total fever duration was longer in the high CRP group than the low CRP group (6.6 ± 2.2 vs 6.3 ± 2.5 days, *p* < 0.001). However, pretreatment fever duration was not significantly different between the two groups (high vs. low group, 5.1 ± 1.7 days vs. 5.2 ± 2.1 days; *p* = 0.206). The principal symptoms, except for Bacillus Calmette–Guérin (BCG) site redness, were all more frequently observed in the high CRP group than in the low CRP group. An incomplete presentation of KD was more frequently observed in the low CRP group than in the high CRP group (high vs. low group, 31.7% vs. 42.1%, *p* < 0.001). White blood cell count, percentage of neutrophil count, aspartate aminotransferase, and alanine aminotransferase levels were significantly higher in the high CRP group than in the low CRP group (Table 1).

**Table 1:** Clinical characteristics between patients with low and high C-reactive protein levels among patient with KD

Characteristics	Total	Low CRP < 3 mg/dL		High CRP ≥ 3 mg/dL		***p***-value
		n	Value	n	Value	
N (%)	9131	2107	23.1	7024	76.9	
Age, months (mean ± SD)	9131	2107	31.7 ± 24.8	7024	34.4 ± 24.9	< 0.001
Male, n (%)	9053	2081	1261 (60.6)	6972	4042 (58.0)	0.033
Weight, kg (mean ± SD)	9131	2107	13.8 ± 6.0	7024	14.2 ± 5.9	0.003
Height, cm (mean ± SD)	9131	2107	90.2 ± 17.4	7024	92.2 ± 17.3	< 0.001
Fever, total days (mean ± SD)	8810	2027	6.3 ± 2.5	6783	6.6 ± 2.2	< 0.001
Fever, pretreatment, days (mean ± SD)	9025	2072	5.2 ± 2.1	6998	5.1 ± 1.7	0.206
Symptoms, n (%)						
Conjunctival injection	9010	2054	1664 (81.0)	6956	6220 (89.4)	< 0.001
Oral changes	9001	2050	1604 (78.2)	6951	5865 (84.4)	< 0.001
Extremity changes	8918	2028	1247 (61.5)	6890	4651 (67.5)	< 0.001
Rash	8984	2053	1484 (72.3)	6931	5349 (77.2)	< 0.001
BCG site redness	8354	1914	769 (40.2)	6440	2520 (39.1)	0.424
Cervical lymphadenopathy	8960	2047	1015 (49.6)	6913	4179 (60.5)	< 0.001
Incomplete presentation, n (%)	8914	2006	844 (42.1)	6908	2187 (31.7)	< 0.001
Family history, n (%)	6543	1414	24 (1.7)	5129	68 (1.3)	0.312
Recurrence, n (%)	8781	2017	99 (4.9)	6763	337 (5.0)	0.953
Laboratory results						
WBC, /mm^3^ (mean ± SD)	9126	2106	11.988 ± 4.881	7020	14.818 ± 5.163	< 0.001
Neutrophil, % (mean ± SD)	9037	2066	52.2 ± 16.9	6971	66.5 ± 14.6	< 0.001
Hemoglobin, g/dL (mean ± SD)	9096	2100	11.6 ± 0.97	6996	11.3 ± 1.02	< 0.001
Platelets, × 10^3^ (mean ± SD)	9105	2103	357.4 ± 126.8	7002	356.5 ± 109.8	0.765
Platelets, ×10 [3], highest (mean ± SD)	8651	2002	422.6 ± 147.0	6649	483.6 ± 165.4	< 0.001
Albumin, g/dL (mean ± SD)	9093	2093	4.0 ± 0.3	7000	3.8 ± 0.4	< 0.001
AST, IU/L (mean ± SD)	9105	2100	64.6 ± 125.9	7005	94.0 ± 173.5	< 0.001
ALT, IU/L (mean ± SD)	9095	2096	59.8 ± 131.5	6999	100.1 ± 149.3	< 0.001
Total bilirubin, mg/dL (mean ± SD)	8978	2070	0.4 ± 0.47	6908	0.68 ± 0.83	< 0.001
Na, mEq/L (mean ± SD)	8983	2059	137.0 ± 2.4	6924	136.2 ± 2.7	< 0.001
CRP, mg/dL (mean ± SD)	9131	2107	1.59 ± 0.86	7024	9.20 ± 5.49	< 0.001
ESR, mm/hr. (mean ± SD)	8356	1919	41.5 ± 24.9	6437	60.7 ± 26.7	< 0.001
BNP, pg/mL (mean ± SD)	2079	519	55.3 ± 94.0	1559	199.4 ± 597.3	< 0.001
ProBNP, pg/mL (mean ± SD)	5533	1266	551.5 ± 1081.5	4267	1624.2 ± 3400.8	< 0.001
Pyuria, n (%)	9008	2076	409 (19.7)	6932	2652 (38.3)	< 0.001

*KD* Kawasaki Disease, *CRP* C-reactive protein, *BCG* Bacillus Calmette–Guérin, *WBC* White blood cell, *AST* Aspartate aminotransferase, *ALT* Alanine aminotransferase, *BNP* B-type natriuretic peptide, *SD* Standard deviation

### Treatment of KD

Overall, 341 (3.7%) patients were not given IVIG because of spontaneous alleviation of fever. Such patients were found more frequently in the low CRP group than in the high CRP group (low vs. high groups, 174 (8.3%) patients vs. 167 (2.4%) patients; *p* < 0.001). The first-line drug treatment for acute KD was IVIG (2 g/kg), and 1602 patients were non-responsive to this treatment (18.3%). The non-response rate was lower in the low CRP group than in the high CRP group (225 [11.7%] patients vs. 1377 [20.1%] patients, respectively; *p* < 0.001). Among patients who were non-responsive to initial IVIG, a second IVIG dose was administered to 1435 patients (89.6%). Among them, 342 patients were non-responsive (23.8%), and there was no significant difference between the two groups (high vs. low group, 302 [24.4%] patients vs. 40 [20.2%] patients; *p* = 0.209; Table 2).

**Table 2:** Response to treatment and coronary artery complications in children with Kawasaki disease

Outcomes	Total n (%)	Low CRP < 3 mg/dL (*n* = 2107), n (%)		High CRP ≥ 3 mg/dL (*n* = 7024), n (%)		***p***-value
Spontaneous remission	341 (3.7)		174 (8.3)		167 (2.4)	< 0.001
1st IVIG	8778 (96.1)		1929 (91.6)		6849 (97.5)	< 0.001
Unresponsive to 1st IVIG	1602/8778 (18.3)		225 (11.7)		1377 (20.1)	< 0.001
2nd IVIG	1435/1602 (89.6)		198 (88.0)		1237 (89.8)	0.453
Unresponsive to 2nd IVIG	342/1435 (23.8)		40 (20.2)		302 (24.4)	0.209
Acute CAL						
z-score	1271/9000 (14.1)	2082	380 (18.3)	6918	891 (12.9)	< 0.001
Japanese criteria	946/9014 (10.5)	2084	260 (12.5)	6930	686 (9.9)	0.001
Acute giant CAA						
z-score	56/9014 (0.6)	2084	13 (0.6)	6930	43 (0.6)	1.0
Japanese criteria	13/9110 (0.1)	2101	3 (0.1)	7009	10 (0.1)	1.0
Convalescent CAL						
z-score	501/8196 (6.1)	1873	160 (8.5)	6323	341 (5.4)	< 0.001
Japanese criteria	419/8203 (5.1)	1875	127 (6.8)	6328	292 (4.6)	< 0.001
Convalescent giant CAA						
z-score	28/8204 (0.3)	1875	1 (0.1)	6329	27 (0.4)	0.011
Japanese criteria	8/8661 (0.1)	1971	0	6690	8 (0.1)	0.212

*KD* Kawasaki Disease, *CRP* C-reactive protein, *IVIG* Intravenous immunoglobulin treatment, *CAL* Coronary artery lesion, *CAA* Coronary artery aneurysm; Japanese criteria, criteria of the Japanese Ministry of Ministry of Health and Welfare

### Coronary artery complications

**Fig. 1:**
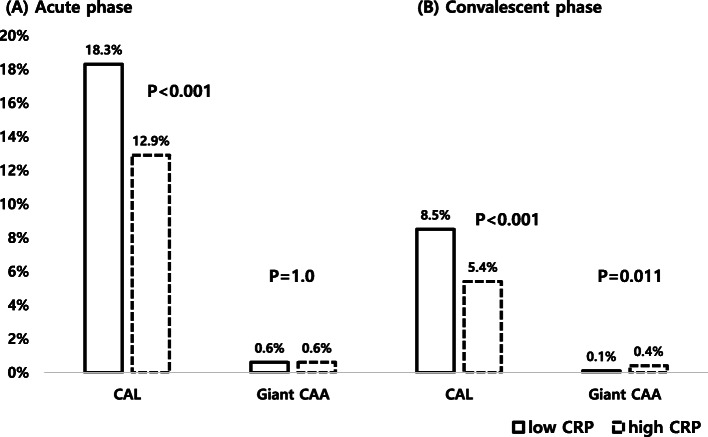
Incidence of coronary artery lesions in Korean children with Kawasaki disease based on the z-score [6]. **A** Acute phase and **B** convalescent phase. CAL; coronary artery lesion, CAA; coronary artery aneurysm, CRP; C-reactive protein

**Fig. 2:**
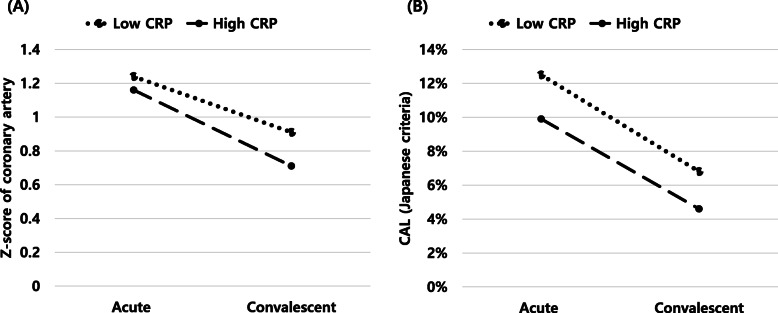
Coronary artery lesion assessment during the acute and convalescence stages in Korean children with Kawasaki disease. A decline in both the size and prevalence of coronary artery lesions in the left main coronary artery during the post-convalescent period was observed compared to the acute phase using both evaluation methods (z-score (**A**) and Japanese criteria [7, 8] (**B**)). CRP; C-reactive protein, CAL; coronary artery lesion

In the acute phase, the overall prevalence of CAL was 14.1% based on the z-score and 10.5% based on the Japanese criteria. After the convalescent period, the CAL prevalence, according to the two criteria, decreased to 6.1 and 5.1%, respectively. A giant CAA was found in 0.6% (z-score) and 0.1% (Japanese criteria) of patients in the acute phase and 0.3% (z-score) and 0.1% (Japanese criteria) after the convalescent period (Fig. 1). A decline in both the size and prevalence of CAL was found in the post-convalescent period compared to the acute phase of KD based on both criteria (z-score and Japanese criteria; Fig. 2).

In this study, the occurrence of CAL was higher in incomplete KD than complete KD. (Supplementary 3), and these incomplete KD was higher in the low CRP group. In the other hand, in an analysis performed only in incomplete KD patients, the occurrence of CAL was frequent in low CRP group than the high CRP group. However in complete KD patients, there was not difference of CAL by CRP group. (Supplementary Table 4A, B).

Fever duration before treatment was one of the risk factors for the development of acute CAL in the multivariable analysis. Regardless of the serum CRP level, if the duration of fever was longer, the risk of CAL was higher (odds ratio [OR], 1.112 [1.064–1.163]; *p* < 0.001). Furthermore, IVIG non-responsiveness was associated with CAL development (OR, 2.617 [2.064–3.317]; *p* < 0.001).

In the high CRP group, the acute CAL risk was higher among non-responders to the second IVIG dose than among non-responders to the first IVIG dose (initial IVIG response: OR, 1.414 [1.223–1.634], p < 0.001; second IVIG response: OR, 2.884 [2.234–3.723], p < 0.001). In the low CRP group, responsiveness to initial IVIG treatment was not associated with CAL occurrence. Increased CAL risk was shown only for non-responders to the second IVIG dose (initial IVIG response: OR, 1.147 [0.811–1.623], *p* = 0.438; second IVIG response: OR, 2.283 [1.162–4.486], *p* < 0.017).

We performed a risk factor analysis for acute CAL development in the low CRP group. In the univariable analysis, the age at diagnosis, incomplete presentation of KD, fever duration before treatment, and non-responsiveness to secondary IVIG showed a statistically significant result (all *p* < 0.05). In the multivariable analysis, all of these factors were also a risk factor for the occurrence of CAL (Table 3).

**Table 3:** Risk factors for coronary artery lesion in patients with low C-reactive protein

	Univariable analysis		Multivariable analysis	
	OR (95% CI)	*p-*value	OR (95% CI)	*p-*value
Age at diagnosis	0.953 (0.901, 1.008)	0.090	0.930 (0.873, 0.991)	0.025
Incomplete KD	2.188 (1.713, 2.794)	< 0.001	2.142 (1.672, 2.745)	< 0.001
Fever, pretreatment	1.092 (1.041, 1.145)	< 0.001	1.101 (1.045, 1.159)	< 0.001
2nd IVIG unresponsiveness	2.283 (1.162, 4.486)	0.017	3.235 (1.550, 6.753)	0.014

During acute phase of KD

*KD* Kawasaki Disease, *OR* Odds ratio, *IVIG* Intravenous immunoglobulin, *BCG* Bacillus Calmette–Guérin

## Discussion

In this retrospective analysis of the 9th Korean nationwide survey of KD, the development of CAL was also found in patients with low serum CRP levels. In the low CRP group, CAL was observed in 18.3 and 12.5% of patients during the acute phase and 8.5 and 6.8% of patients after the convalescent period based on the z-score and Japanese criteria, respectively.

CAL occurrence was higher in the low CRP group than in the high CRP group. Our study design did not allow us to identify or define the cause of this observation. However, patients in the low CRP group were younger at KD diagnosis and more often presented with incomplete KD, which may be associated with a higher CAL occurrence than in patients in the high CRP group [1, 9, 10]. In analysis of incomplete KD, the occurrence of CAL was higher in low CRP group than that in high CRP group, but this pattern was not prominent in analysis of complete KD. Therefore, it should be noted that CAL may occur even in incomplete patients with low CRP.

Although we did not describe it above, the patients have been also evaluated by ESR level (low ESR < 40 mm/hr. ≤ high ESR) (supplementary Table 1). As the result of CRP, it was confirmed that CAL occurred even in the low ESR group. In other words, it was confirmed that CAL also occurred in patients with low initial inflammatory maker level.

In general, the risk assessment of coronary artery complications is evaluated based on IVIG responsiveness, and high serum CRP levels are regarded as a related factor for the non-responsiveness to IVIG [11, 12]. The CRP value used to predict this non-responsiveness to IVIG is the initial CRP level, which reflects the inflammatory state prior to IVIG treatment. However, the initial serum CRP level alone may not be sufficient to predict complications. Nandi et al. showed a difference in the CRP and interleukin-6 (IL-6; a cytokine that stimulates inflammatory markers, including CRP) levels between IVIG responders and non-responders. They found that CRP and IL-6 levels were higher after IVIG treatment in IVIG non-responders than in responders; however, these levels were not significantly different before IVIG treatment [5]. High CRP levels after initial IVIG were also reported to be a risk factor for non-responsiveness to additional IVIG treatment in refractory KD patients [13, 14]. Because the serum CRP levels after IVIG treatment were not investigated in this study, it is difficult to draw definitive conclusions, but we believe that the serum CRP level before IVIG treatment is insufficient to predict coronary artery complications.

In our study, the fever duration before treatment was not different between the low and high CRP groups, but there was a difference in responsiveness to IVIG treatment. In patients with low serum CRP levels, the rate of non-responsiveness to the first IVIG dose was 11.7%, which was considerably lower than the overall nonresponse rate (18.3%) to the first IVIG dose among all patients. In patients with low serum CRP levels, the response rate to the first IVIG dose was good, but coronary artery complications still occurred in some of these patients, which suggest that patients with low CRP levels may not be a homogeneous group. Some patients responded well to treatment without coronary complications, as would be expected based on their low CRP serum levels in others, coronary complications were found although they had appropriate treatment. One may hypothesize that a rise in CRP levels occurred after the initial test, but further research is required to test such a hypothesis.

In both groups, the presence of some principle symptoms was associated with a low risk of CAL. Notably, in the low CRP group, the presence of BCG site redness showed a low risk of CAL. Similar trends were identified in the high CRP group, with the presence of principle symptoms associated with a low risk of CAL. These results are in line with the findings of a previous study, which showed that coronary artery complications may be high due to the delayed diagnosis and treatment of patients with incomplete KD who have insufficient clinical symptoms to match the diagnostic criteria [1].

In this study, the characteristics of patients were similar to those of patients in the nationwide survey and other reported data [15–17]. However, our results should be interpreted within the limitations of this study. Because this analysis was performed retrospectively, patients lacking essential data required to interpret the results of this study were excluded (*n* = 6256); thus, 9131 patients who met the inclusion criteria were analyzed. Furthermore, serum CRP levels were collected just prior to initial IVIG treatment, but the exact day of sampling from fever onset was not investigated. The serum CRP level was associated with KD disease duration and it could not be linked with coronary artery outcome directly. In addition, since CRP after IVIG treatment has not been investigated, we cannot make any conclusions about the occurrence of CALs following CRP changes.

## Conclusions

Coronary artery complications in KD occurred not only with high serum CRP levels (≥3 mg/dL) but also low serum CRP levels (< 3 mg/dL) of before IVIG treatment. In addition, the CAL occurrence rate was higher in patients with low serum CRP levels than in patients with high serum CRP levels, which was associated with the higher CAL occurrence rate in incomplete KD. Therefore, we recommend monitoring the clinical course of KD carefully, regardless of the serum CRP level, and to note that coronary artery complications are not predictable based on serum CRP levels prior to IVIG treatment alone.

## Supplementary Information

**Additional file 1.** Coronary artery complications in children with Kawasaki disease (ESR).

**Additional file 2.** Coronary artery complications in children with Kawasaki disease (right coronary artery).

**Additional file 3.** Coronary artery complications in children with Kawasaki disease (complete vs. incomplete).

**Additional file 4: Supplementary 4A**. Coronary artery complications in children with Kawasaki disease (incomplete KD). **Supplementary 4B**. Coronary artery complications in children with Kawasaki disease (complete KD).

## Data Availability

The datasets used and/or analysed during the current study are available from the corresponding author on reasonable request.

## Abbreviations

BCG: Bacillus Calmette-Guerin
CAA: Coronary artery aneurysm
CAL: Coronary artery lesions
CRP: C-reactive protein
IVIG: Intravenous immunoglobulin
KD: Kawasaki Disease
