# Supplementary material for: The occurrence of coronary artery lesions in Kawasaki disease based on C-reactive protein levels: a retrospective cohort study
Source: Pediatr Rheumatol Online J. 2021 Jun 2;19:78. doi: 10.1186/s12969-021-00566-6 (PMC8173749; doi:10.1186/s12969-021-00566-6)
Supplement: Supplementary file 1 — Additional file 1. Coronary artery complications in children with Kawasaki disease (ESR). [file 12969_2021_566_MOESM1_ESM.docx]

Coronary artery complications in children with Kawasaki disease (ESR)

| **Outcomes** | **Total, 8356**  　n (%) | **ESR < 40 mm/hr**  (n = 2436), n (%) | | **ESR, ≥ 40 mm/hr**  (n = 5920), n (%) | | **p-value** |
| --- | --- | --- | --- | --- | --- | --- |
| Acute CAL |  |  |  |  |  |  |
| z-score | 1162/8234 (14.1) | 2042 | 378 (15.8) | 5832 | 783 (13.4) | 0.006 |
| Japanese criteria | 873/8247 (10.6) | 2405 | 237 (9.9) | 5842 | 636 (10.9) | 0.168 |
| Acute giant CAA |  |  |  |  |  |  |
| z-score | 53/8194 (0.6) | 2405 | 12 (0.5) | 5842 | 41 (0.7) | 0.363 |
| Japanese criteria | 12/8336 (0.1) | 2430 | 4 (0.2) | 5906 | 8 (0.1) | 0.755 |
| Convalescent CAL |  |  |  |  |  |  |
| z-score | 463/7501 (6.2) | 2218 | 167 (7.5) | 5283 | 296 (5.6) | 0.002 |
| Japanese criteria | 393/7507 (5.2) | 2220 | 121 (5.5) | 5287 | 272 (5.1) | 0.609 |
| Convalescent giant CAA |  |  |  |  |  |  |
| z-score | 27/7508 (0.4) | 2220 | 4 (0.2) | 5288 | 23 (0.4) | 0.137 |
| Japanese criteria | 8/7924(0.1) | 2319 | 1 (0.0) | 5605 | 7 (0.1) | 0.451 |
