# Supplementary material for: The occurrence of coronary artery lesions in Kawasaki disease based on C-reactive protein levels: a retrospective cohort study
Source: Pediatr Rheumatol Online J. 2021 Jun 2;19:78. doi: 10.1186/s12969-021-00566-6 (PMC8173749; doi:10.1186/s12969-021-00566-6)
Supplement: Supplementary file 2 — Additional file 2. Coronary artery complications in children with Kawasaki disease (right coronary artery). [file 12969_2021_566_MOESM2_ESM.docx]

Supplementary 2. Coronary artery complications in children with Kawasaki disease (right coronary artery)

| **Outcomes** | **Total, 9131**  　n (%) | **Low CRP < 3 mg/dL**  (n = 2107), n (%) | | **High CRP ≥ 3 mg/dL**  (n = 7024), n (%) | | **p-value** |
| --- | --- | --- | --- | --- | --- | --- |
| Acute CAL |  |  |  |  |  |  |
| z-score | 672/8904 (7.5) | 2054 | 103 (5.0) | 6850 | 569 (8.3) | <0.001 |
| Japanese criteria | 373/8917 (4.2) | 2057 | 64 (3.1) | 6860 | 309 (4.5) | 0.006 |
| Acute giant CAA |  |  |  |  |  |  |
| z-score | 36/8915 (0.4) | 2057 | 7 (0.3) | 6858 | 29 (0.4) | 0.696 |
| Japanese criteria | 5/8918 (0.1) | 2057 | 1 (0.0) | 6861 | 4 (0.1) | 1.0 |
| Convalescent CAL |  |  |  |  |  |  |
| z-score | 248/8122 (3.1) | 1854 | 42 (2.3) | 6268 | 206 (3.3) | 0.026 |
| Japanese criteria | 106/8125 (1.3) | 1854 | 15 (0.8) | 6328 | 91 (1.5) | 0.035 |
| Convalescent giant CAA |  |  |  |  |  |  |
| z-score | 18/8124 (0.2) | 1854 | 1 (0.1) | 6270 | 17 (0.3) | 0.094 |
| Japanese criteria | 5/8131 (0.1) | 1854 | 0 (0.0) | 6277 | 5 (0.1) | 0.595 |
