# Supplementary material for: The occurrence of coronary artery lesions in Kawasaki disease based on C-reactive protein levels: a retrospective cohort study
Source: Pediatr Rheumatol Online J. 2021 Jun 2;19:78. doi: 10.1186/s12969-021-00566-6 (PMC8173749; doi:10.1186/s12969-021-00566-6)
Supplement: Supplementary file 3 — Additional file 3. Coronary artery complications in children with Kawasaki disease (complete vs. incomplete). [file 12969_2021_566_MOESM3_ESM.docx]

Supplementary 3. Coronary artery complications in children with Kawasaki disease (complete vs. incomplete)

| **Outcomes** | **Total, 9131**  　n (%) | **Complete KD**  (n = 5883), n (%) | | **Incomplete KD**  (n = 3031), n (%) | | **p-value** |
| --- | --- | --- | --- | --- | --- | --- |
| Acute CAL |  |  |  |  |  |  |
| z-score | 1145/8790 (13.0) | 5808 | 623 (10.7) | 2982 | 522 (17.5) | <0.001 |
| Japanese criteria | 852/8804 (9.7) | 5816 | 485 (8.3) | 2988 | 367 (12.3) | <0.001 |
| Convalescent CAL |  |  |  |  |  |  |
| z-score | 449/8010 (5.6) | 5355 | 220 (4.1) | 2655 | 229 (8.6) | <0.001 |
| Japanese criteria | 376/8017 (4.7) | 5359 | 210 (3.9) | 2658 | 166 (6.2) | <0.001 |
