# Supplementary material for: The occurrence of coronary artery lesions in Kawasaki disease based on C-reactive protein levels: a retrospective cohort study
Source: Pediatr Rheumatol Online J. 2021 Jun 2;19:78. doi: 10.1186/s12969-021-00566-6 (PMC8173749; doi:10.1186/s12969-021-00566-6)
Supplement: Supplementary file 4 — Additional file 4: Supplementary 4A. Coronary artery complications in children with Kawasaki disease (incomplete KD). Supplementary 4B. Coronary artery complications in children with Kawasaki disease (complete KD). [file 12969_2021_566_MOESM4_ESM.docx]

Supplementary 4A. Coronary artery complications in children with Kawasaki disease (incomplete KD)

| **Outcomes** | **Total, 3031**  　n (%) | **Low CRP < 3 mg/dL**  (n = 844), n (%) | | **High CRP ≥ 3 mg/dL**  (n = 2187), n (%) | | **p-value** |
| --- | --- | --- | --- | --- | --- | --- |
| Acute CAL |  |  |  |  |  |  |
| z-score | 522/2982 (17.5) | 830 | 183 (22) | 2152 | 339 (15.8) | <0.001 |
| Japanese criteria | 367/2988 (12.3) | 831 | 120 (14.4) | 2157 | 247 (11.5) | 0.029 |
| Convalescent CAL |  |  |  |  |  |  |
| z-score | 229/2655 (8.6) | 738 | 91 (12.3) | 1917 | 138 (7.2) | <0.001 |
| Japanese criteria | 166/2658 (6.2) | 739 | 63 (8.5) | 1919 | 103 (5.4) | 0.001 |

Supplementary 4B. Coronary artery complications in children with Kawasaki disease (complete KD)

| **Outcomes** | **Total, 5883**  　n (%) | **Low CRP < 3 mg/dL**  (n = 1162), n (%) | | **High CRP ≥ 3 mg/dL**  (n = 4721), n (%) | | **p-value** |
| --- | --- | --- | --- | --- | --- | --- |
| Acute CAL |  |  |  |  |  |  |
| z-score | 623/5808 (10.7) | 1153 | 132 (11.4) | 4655 | 491 (10.5) | 0.395 |
| Japanese criteria | 485/5618 (8.3) | 1154 | 95 (8.2) | 4662 | 390 (8.4) | 0.953 |
| Convalescent CAL |  |  |  |  |  |  |
| z-score | 220/5355 (4.1) | 1047 | 41 (3.9) | 4308 | 179 (4.2) | 0.795 |
| Japanese criteria | 210/5359 (3.9) | 1048 | 43 (4.1) | 4311 | 167 (3.9) | 0.723 |
